# Shaping the future of human biomonitoring (HBM): progress, strategy, and global vision from ISES Europe and the HBM Global Network^☆^

**DOI:** 10.1016/j.envint.2025.109985

**Published:** 2025-12-07

**Authors:** Maryam Zare Jeddi, Nancy B. Hopf, Karen S. Galea, Kate Jones, Henriqueta Louro, Maria João Silva, Adrian Covaci, Tiina Santonen, Paul T.J. Scheepers, Susana Viegas, Lesliam Quirós-Alcalá, Asif Qureshi, M. Elizabeth Marder, Natalie von Goetz, Konstantinos M. Kasiotis, Kyriaki Machera, Ovnair Sepai, Radu-Corneliu Duca, Manosij Ghosh, An van Nieuwenhuyse, Ming Kei Chung, Jihyon Kil, Shoji F. Nakayama, Aziza Menouni, Kaoutar Chbihi, Ana Maria Vekic, Gustavo Souza, Maisarah Nasution Waras, Imran Ali, Michael Bader, Eva Kumar, Konstantinos C. Makris, Elizabeth Ziying Lin, Erin N. Haynes, Yu Ait Bamai, Jung-Hwan Kwon, Po-Chin Huang, Robert Pasanen-Kase

**Affiliations:** a Shell Global Solutions Internationals BV, 2596HR Den Haag, The Netherlands; b Unisanté, University Center for Primary Care and Public Health & University of Lausanne, Lausanne 1010, Switzerland; c Institute of Occupational Medicine (IOM), Edinburgh, EH14 4AP, United Kingdom; d Health and Safety Executive (HSE), Buxton SK17 9JN, United Kingdom; e Department of Human Genetics, National Institute of Health Doutor Ricardo Jorge, 1649-016 Lisbon, Portugal; f Comprehensive Health Research Center, CHRC, NOVA Medical School, NOVA University of Lisbon, 1150-082 Lisbon, Portugal; g Toxicological Center, University of Antwerp, Universiteitsplein 1, 2610 Wilrijk, Belgium; h Finnish Institute of Occupational Health (FIOH), Helsinki, Finland; i Radboud Institute for Biological and Environmental Sciences, Radboud University, Nijmegen, The Netherlands; j NOVA National School of Public Health, Public Health Research Centre, Comprehensive Health Research Center, CHRC, REAL, CCAL, NOVA University Lisbon, Lisbon, Portugal; k Department of Environmental Health and Engineering, Johns Hopkins University Bloomberg School of Public Health, Baltimore, MD, USA; l Department of Climate Change & Department of Civil Engineering, IIT Hyderabad, Kandi, Sangareddy, Telangana 502285 India; m Department of Environmental Toxicology, University of California, Davis, Davis, CA, USA; n Federal Office of Public Health, Bern, Switzerland; o Laboratory of Pesticides’ Toxicology, Scientific Directorate of Pesticides Control and Phytopharmacy, Benaki Phytopathological Institute,14561 Kifissia, Athens, Greece; p Toxicology Department, Radiation, Chemicals, Climate and Environmental Hazards Division, UK Health Security Agency, United Kingdom; q Department of Health Protection, National Health Laboratory (LNS), L-3555 Dudelange, Luxembourg; r Environment and Health Unit, Department of Public Health and Primary Care, Katholieke Universiteit Leuven (KU Leuven), 3000 Leuven, Belgium; s The Jockey Club School of Public Health and Primary Care, The Chinese University of Hong Kong, Hong Kong, China; t The Institute of Environment, Energy and Sustainability, The Chinese University of Hong Kong, Hong Kong, China; u Li Ka Shing Institute of Health Sciences, The Chinese University of Hong Kong, Hong Kong, China; v Department of Earth and Environmental Sciences, The Chinese University of Hong Kong, Hong Kong, China; w Environmental Health Research Division, National Institute of Environmental Research, Ministry of Climate, Energy and Environment, Incheon 22689, the Republic of Korea; x Exposure Dynamics Research Section, Health and Environmental Risk Division, National Institute for Environmental Studies, 16-2 Onogawa, Tsukuba, Ibaraki 305-8506, Japan; y Department of Research, Thriving Lab, 20000 Casablanca, Morocco; z Human Epidemiology and Environmental Health Research Team, Moulay Ismail University of Meknes 50000 Meknes, Morocco; aa Department of Environmental and Occupational Health Surveillance, Secretariat of Health and Environment Surveillance, Ministry of Health, Brazil; ab Department of Toxicology, Advanced Medical and Dental Institute, Universiti Sains Malaysia, 13200 Kepala Batas, P. Pinang, Malaysia; ac Swedish Chemicals Agency, Department of Authorisation/Health Assessment, Stockholm, Sweden; ad BASF SE, Corporate Health Management, 67056 Ludwigshafen, Germany; ae Department of Environmental Technology, Savonia University of Applied Sciences, FI-70201 Kuopio, Finland; af Cyprus International Institute for Environmental and Public Health, School of Health Sciences, Cyprus University of Technology, 3041 Limassol, Cyprus; ag Environmental Health Sciences Department, School of Public Health, Yale University, New Haven, Connecticut 06510, United States; ah Department of Epidemiology and Environmental Health, College of Public Health, University of Kentucky, Lexington, KY 40536, United States; ai Center for Environmental and Health Sciences, Hokkaido University, Kita 12, Nishi 7, Kita-ku, Sapporo, Japan; aj Division of Environmental Science and Ecological Engineering, Korea University, Seoul 02841, Republic of Korea; ak National Institute of Environmental Health Sciences, National Health Research Institutes, Miaoli 35053, Taiwan, ROC; al Department of Medical Research, China Medical University Hospital, China Medical University, Taichung 404327, Taiwan, ROC; am State Secretariat for Economic Affairs SECO, Section Chemicals and Occupational Health, 3003 Bern, Switzerland

**Keywords:** Human biomonitoring, Exposure science, Harmonization, FAIR data, Global community, Data quality

## Abstract

Human biomonitoring (HBM) continues to play an indispensable role within exposure science, offering insights into aggregate chemical exposures across populations and life stages. Since 2018, the European chapter of the International Society of Exposure Science Human Biomonitoring Working Group (ISES Europe HBM WG) has aimed to facilitate generation of more and high-quality HBM data. The working group aims to strengthen integration of HBM data into regulatory frameworks through improved study design, harmonized methodologies, and enhanced reporting practices. Key achievements in the past seven years include the harmonization of HBM metadata through development of minimum information requirements for HBM (MIR-HBM), development of chemical-specific BASIC Guides for occupational health and hygiene professionals, and establishment of the FAIR (Findable, Accessible, Interoperable, and Reusable) Environmental and Health Registry (FAIREHR) to enhance data transparency and reusability. Recognizing the need for broader impact, the HBM Global Network was launched in 2025 to promote worldwide collaboration, capacity building, and policy integration. Together, ISES Europe HBM WG and the HBM Global Network form a coordinated platform with shared governance, strategic priorities, and digital infrastructure. This short communication outlines the progress to date, strategic pillars guiding our work, and ongoing initiatives linking science, policy, and practice. We call on researchers, regulators, and stakeholders worldwide to join these networks, strengthen harmonized approaches, and ensure that HBM becomes a cornerstone of 21st-century chemical risk governance.

## 1. Introduction

Human biomonitoring (HBM) is a cornerstone of exposure science, enabling the assessment of internal and aggregate chemical exposures from multiple sources and routes across populations and life stages (Lamon et al., 2024; Santonen et al., 2023; Reale et al., 2024). HBM has gained growing attention as an essential approach in understanding human exposure to chemicals, their impact on human health and informing regulatory decision-making (Viegas et al., 2024; WHO, 2023b). Major national and international programmes, such as the EU projects Human Biomonitoring for the Europe (HBM4EU) (Gilles et al., 2021) and the Partnership for the Assessment of Risks from Chemicals (PARC) (Marx-Stoelting et al., 2023), Canada’s national HBM programme through the Canadian Health Measures Survey (CHMS) (HealthCanada, 2024), and initiatives led by the Organisation for Economic Co-operation and Development (OECD) (OECD, 2022; OECD 2025a; OECD 2025b), have demonstrated the value of HBM data for public and occupational health protection. The lack of standardization and alignment between independent studies, regulatory frameworks, and research initiatives continues to pose challenges for fully leveraging HBM potentials. Experts across the globe need to keep track of activities, integrate their findings, and build on existing methodologies rather than working in silos. Currently, many research and monitoring efforts operate in isolation, leading to potential duplication, inefficient resource use, and missed opportunities for coordinated action. Addressing these challenges requires harmonized methodologies, structured collaboration, and an integrated platform to leverage existing work and expertise.

The European chapter of the International Society of Exposure Science Human Biomonitoring Working Group (ISES Europe HBM WG) was established in 2018 to address these challenges. The ISES Europe HBM WG’s mission is to unify and coordinate expertise, generate high quality and harmonized HBM data, and bridge the gap between research, policy, and practice (Zare Jeddi et al., 2022b).

## 2. Strategic pillars of the ISES Europe HBM WG

**Fig. 1.**
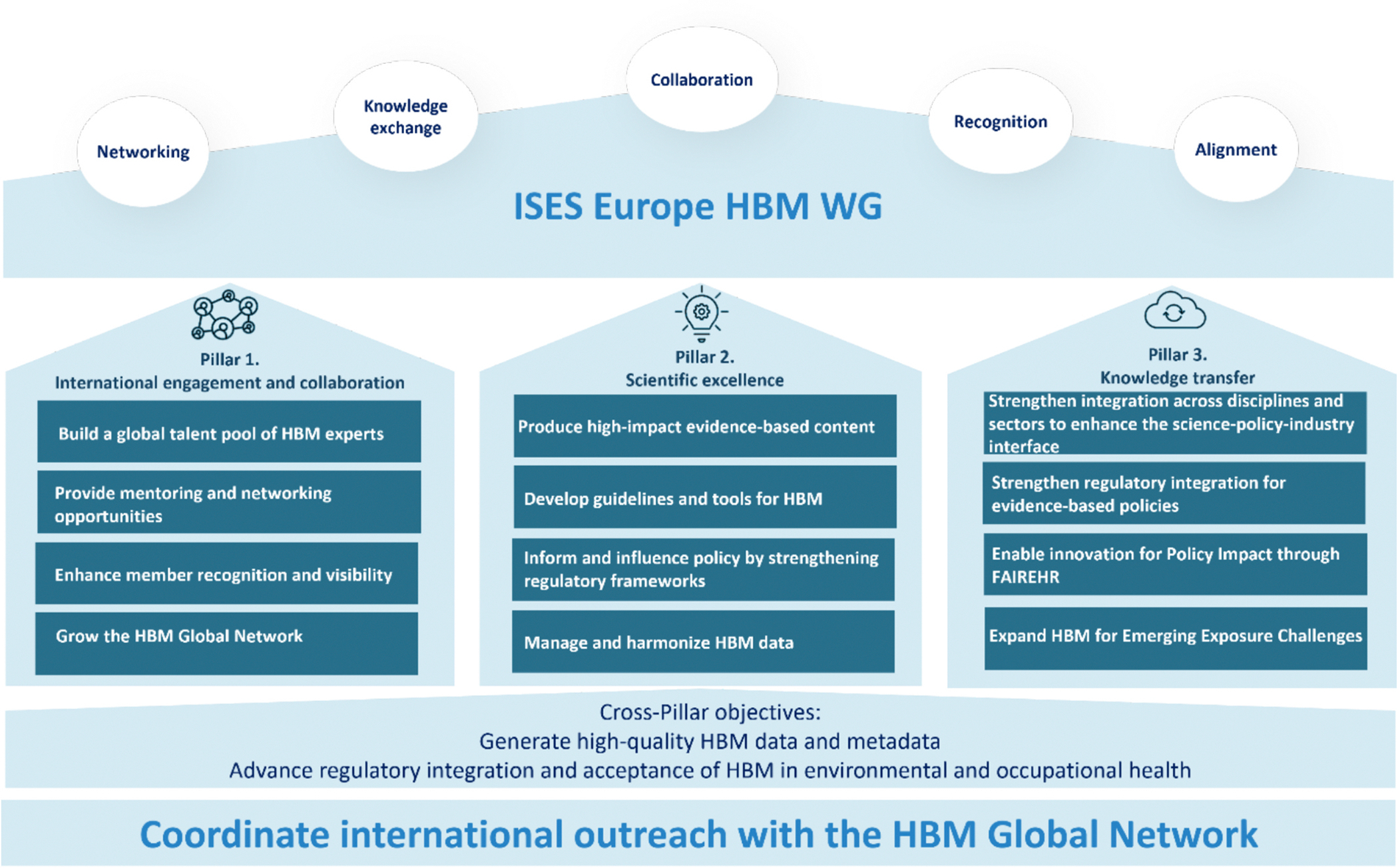
ISES Europe HBM WG global strategic priorities to enhance the impact and use of human biomonitoring (HBM).

The ISES Europe HBM WG is structured around three strategic pillars to fulfill its mission (Fig. 1).

The first pillar focuses on **enabling international engagement and collaboration**. Specifically, it aims to build a global network of skilled experts, offering mentoring, peer-support, and collaboration opportunities. This approach promotes inclusiveness and enhances member recognition and visibility.

The second pillar focuses on **advancing scientific excellence**, supported by three objectives: (1) proactively pursuing impactful scientific content, (2) supporting the development and application of guidance, methods, and tools to enhance the use of HBM, and (3) fostering connectivity across scientific disciplines. This includes raising awareness of exposure science and promoting its use as a key approach in exposure assessment tools across other disciplines. The third pillar emphasizes **enhancing knowledge transfer for evidence-based policies**. We envision the HBM WG and the HBM Global Network as knowledge translation hubs, effectively communicating scientific advancements to broad and diverse audiences and, most importantly, leveraging data for evidence-based policies and regulations, where HBM can play a crucial role.

Some achievements of the HBM WG may span across multiple strategic pillars, highlighting their broader impact and interdisciplinary nature.

Future initiatives for each topic will be made available on the dedicated webpage of the HBM WG, accessible via the ISES Europe website (https://ises-europe.org/group/human-biomonitoring). The activities of the ISES Europe HBM WG are presented in various relevant conferences, such as International Occupational Hygiene Association (IOHA), ISBM (International Symposium on Biological Monitoring), ISES (International Society of Exposure Science), ISEE (International Society for Environmental Epidemiology), among others. In addition, ISES Europe holds annual meetings that feature a dedicated session for the HBM WG, where the WG members evaluate the ongoing work, discuss progress and collect information for further improvement, initiatives and development.

## 3. Achievements and impacts to date by strategic pillars

### 3.1. Pillar 1. International engagement and collaboration: Scaling our impact

**Fig. 2.**
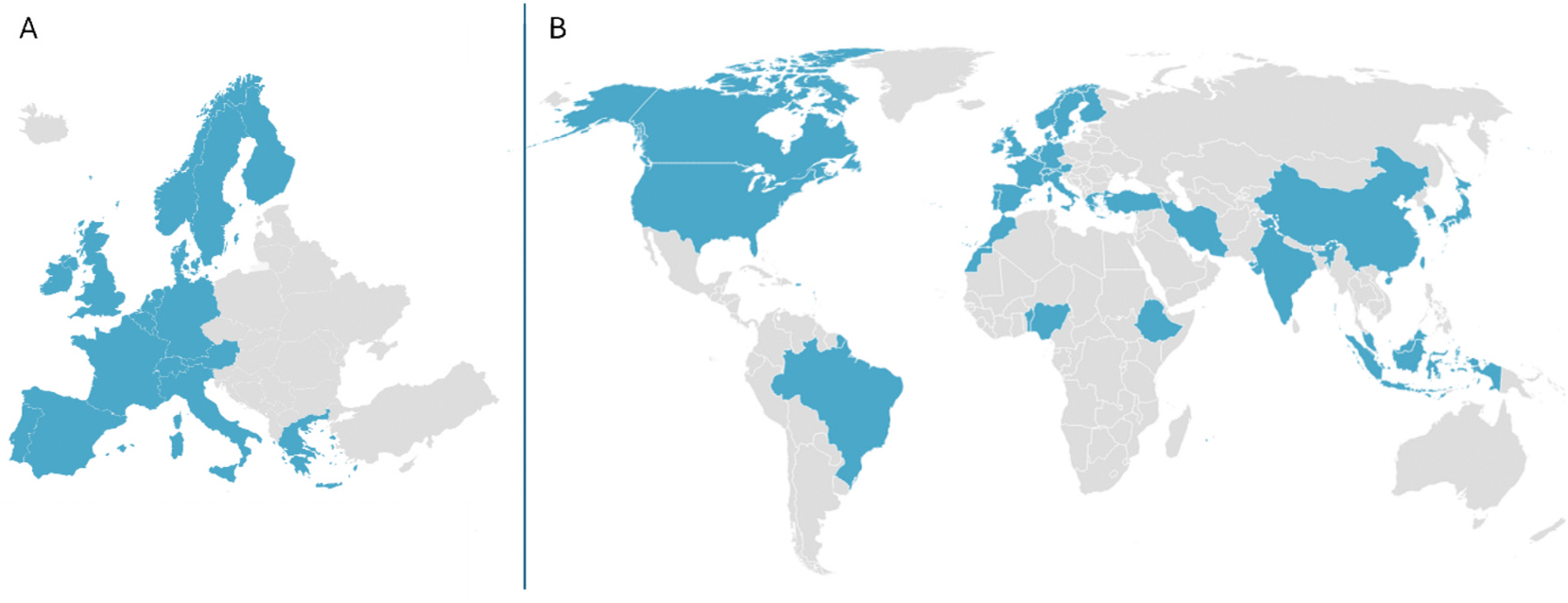
A. geographical coverage (17 countries) of ISES europe (pan-european) human biomonitoring working group (ISES Europe HBM WG) in 2018.Fig. 2b. Geographical coverage (34 countries) of the Human biomonitoring (HBM) Global Network in 2025.

In 2018, ISES Europe established a dedicated HBM Working Group (ISES Europe HBM WG), bringing together approximately 40 experts from academia, research institutions, regulatory agencies, and industry across Europe (Fig. 2a). The group embodies a wide range of disciplines, including exposure science, occupational hygiene, environmental epidemiology, analytical chemistry, risk assessment, toxicology, and public health policy. This vibrant interdisciplinary community is united by a shared commitment to advancing the science and practice of HBM in Europe. Its collective efforts focus on harmonizing methodologies, improving data quality and comparability, and strengthening the integration of HBM into regulatory frameworks and public health strategies.

The ISES Europe HBM WG was established with a European focus, yet its vision and activities resonate with the global need for harmonized HBM, contributing to international collaboration and shared standards. Recognizing that chemical exposures transcend borders with potential impacts on human health (Duarte-Davidson et al., 2014; Hague et al., 2021), the ISES Europe HBM WG has taken a proactive role in fostering international collaboration. This includes the development of the HBM Global Network (https://www.fairehr.com/HBMGlobalNetworks), a dedicated initiative (or coalition) that connects experts, projects, and institutions worldwide to advance data quality, comparability, and policy relevance in HBM. This solution addresses the need for inclusive and sustained collaboration across regions. Developing and conducting HBM studies in certain low- and middle-income countries (LMICs) entails challenges including limited resources, regulatory hurdles, and ethical approval processes. These challenges are not unique to HBM and are common across many public health initiatives. However, HBM has specific requirements, including knowledge of occupational and environmental exposures, sampling logistics, and laboratory capacity that can represent additional challenges. The HBM Global Network helps address these barriers by facilitating knowledge exchange (associated with pillar 3 presented below), peer support, and capacity building across regions. Currently, the HBM Global Network includes over 70 experts from multiple continents (Fig. 2b), reflecting its inclusive and interdisciplinary nature. The HBM Global Network is expanding its outreach to experts in underrepresented regions, including Australia, New Zealand, Latin America, Africa, Asia, Middle East, and the Caribbean (Box 1). These regions are currently less represented in the Network (Fig. 2b), yet they face documented gaps in sustained national HBM capacity. At the same time, emerging policy windows and regional platforms offer promising opportunities to accelerate harmonized implementation (Tamayo-Ortiz et al., 2022; UNEP, 2025). By engaging with these regions, the HBM Global Network aims to foster inclusive collaboration, support capacity building, and strengthen the global relevance of HBM.

Our international engagement to date has built on strong foundations, including partnerships with the ISES International Human Biomonitoring Guidance Value (i-HBM) working group (Nakayama et al., 2023) and capacity-building initiatives, such as the BioNet project (https://bionet-project.org/).

The ISES i-HBM working group focuses on promoting the health-based interpretation of HBM data. The ISES i-HBM working group, with members in 18 countries (of which 6 are outside Europe), developed the first curated database of Human Biomonitoring Health-Based Guidance Values (HB2GVs) in the freely accessible HB2GV Dashboard (https://www.intlexposurescience.org/i-hbm/) (Nakayama et al., 2023).

While HBM implementation in LMICs is challenging, it is feasible and scalable with strategic investment in training, infrastructure, and international collaboration. The HBM Global Network can play a key role in facilitating North-South partnerships, sharing protocols, and supporting ethical and regulatory navigation. The BioNet project (accessible at: https://bionet-project.org/) is a model for sustainable capacity building. BioNet, funded under the EU Erasmus + program, has fostered collaboration between European (Belgium, Luxembourg, Denmark) and African (Morocco, Benin, Ethiopia) universities and public health institutions, strengthening skills in environmental health, biomonitoring, and occupational health surveillance. It has also piloted a scalable e-learning platform and established a sentinel surveillance system for hazardous chemical exposures in Africa. While BioNet concludes in 2025, it provides a blueprint for expanding global engagement beyond Europe-led projects. Moreover, the project will be leading the creation of an African Network for HBM, to be established by 2026, and which will be endorsed by the HBM global network.

With a vision to unite efforts across borders toward a shared goal, the HBM Global Network promotes inclusive governance and digital coordination through FAIREHR (Findable, Accessible, Interoperable, and Reusable Environmental and Health Registry). This framework supports the growing collaboration and helps address global challenges related to chemical exposure. FAIREHR is a global initiative for preregistering HBM studies and programs in exposure science and environmental epidemiology, ensuring that data that is Findable, Accessible, Interoperable, and Reusable (FAIR). By promoting transparency, reproducibility, and policy relevance, FAIREHR strengthens the impact of HBM and fosters trust across scientific and regulatory communities (Galea et al., 2025; Zare Jeddi et al., 2023).

The HBM Global Network will implement a post 2025 global capacity building strategy to achieve the 2030 targets of 25% increase in the number of countries represented in the network, increased adoption of harmonized HBM metadata and use of FAIREHR platform, and the development of at least 15 new BASIC Guides (Human Biomonitoring and Surveillance of Chemical Exposure in Occupational Settings). The BASIC Guides are concise, chemical specific protocols supporting the interpretation and use of HBM in exposure and risk assessment and managing workplace chemical exposure (Zare Jeddi et al., 2026; Hopf et al., 2026).

This strategy will include:

- **Regional HBM Hubs**: Establish centers of excellence in Africa, Asia-Pacific, Latin America, and the Middle East to serve as focal points for robust HBM design, laboratory support, and policy dialogue.
- **Training programs**: Deploy adaptable HBM training curricula to create a multiplier effect in local capacity building, enabling sustainable knowledge transfer and skill development.
- **Digital Collaboration** and Data Tools: Expand FAIREHR functionalities to include multilingual support, virtual mentoring, and open access technical resources, enhancing global accessibility and coordination.
- **Partnership Integration**: Continue collaborations with WHO (World Health Organization) and OECD, and initiate collaboration with UNEP (United Nations Environment Programme), ILO (International Labour Organization), and regional public health agencies to align monitoring priorities, ensure policy relevance, and secure co-funding for sustainability. While these organizations play a critical role in promoting HBM at the policy and governmental level, often through regional strategies and country-specific guidance, the HBM Global Network focuses on operationalizing HBM by sharing expertise, tools, and real-world experience (Box 2).

This approach ensures that international engagement moves from ad hoc project-based collaborations to a permanent, globally distributed network capable of sustaining harmonized biomonitoring well beyond 2030. By embedding capacity building in multiple regions and integrating with global policy frameworks, the HBM Global Network will become a durable backbone for coordinated chemical exposure assessment and risk governance worldwide.

### 3.2. Pillar 2. Advancing scientific excellence

The activities of the ISES Europe HBM WG in advancing scientific excellence are around two overarching goals: 1) generate high-quality HBM data and 2) enhance the regulatory use of HBM data. Based on these overarching goals, in 2022, our WG proposed a strategic roadmap for 2020–2030, emphasizing advancements in HBM sampling methodologies, analytical techniques, harmonization of data, sustainable funding mechanisms, legislative integration, and enhanced stakeholder communication (Zare Jeddi et al., 2022b). In line with the defined strategic objectives to achieve the overarching goals, a series of initiatives were implemented by the ISES Europe HBM WG, often in collaboration with international partners, projects, and agencies. These initiatives align with the strategic objectives and demonstrate concrete impacts on both the scientific and policy communities (Table 1).

### 3.3. Pillar 3: Enhancing knowledge transfer

A core mandate of the ISES Europe HBM WG and the HBM Global Network is to ensure that high-quality scientific evidence translates into effective public health, occupational safety, and environmental policies. Our activities promote the use of HBM data in regulatory frameworks to support evidence-based decision-making. Strengthening regulatory integration is about making sure HBM data does not just stay in scientific reports, but actively shape evidence-based policies that protect public health. This includes aligning biomonitoring outputs with chemical safety evaluations, risk assessments, and policy development processes at national and international levels. Additionally, by operating at the science-policy-industry interface, we aim to translate HBM scientific evidence into actionable insights that support regulatory decision-making and industrial best practices. A key component of the HBM Global Network’s strategy is also to foster Environmental Health Literacy among stakeholders and the broader public. Environmental Health Literacy refers to the ability to understand and act upon information about environmental exposures and their health implications (Hoover, 2018; Lin et al., 2025; Lindsey et al., 2021). Environmental Health Literacy can help individuals make informed decisions about their health, such as avoiding exposure to environmental toxins and air pollutant (Raufman et al., 2020). By promoting Environmental Health Literacy, the HBM Global Network empowers individuals and communities to interpret biomonitoring data, recognize environmental health risks, and engage in informed decision-making. This leads to more effective risk communication, public engagement, and policy development, ensuring that scientific insights are translated into meaningful exposure prevention and health protection measures. This can be achieved by targeted training, the co-creation of communication materials, transparent discussion of uncertainties, and collaboration with local health professionals and educators. Three key activities in this pillar include:

#### a. Science-policy-Industry interface

Our activities can contribute to:

- EU Chemicals Strategy for Sustainability: for instance, by providing population-level exposure data to guide substitution of hazardous chemicals and supporting HBM integration into OSOA (One Substance One Assessment) approach as recently gained attention in the Council and Parliament’s provisional package.^1^
- REACH Regulation: for instance, by supplying biomonitoring evidence for restriction dossiers, chemical safety report evaluations, and post-restriction effectiveness assessments.
- Zero Pollution Action Plan: for instance, by enabling progress tracking toward pollutants’ reduction targets.
- WHO Chemicals Road Map^2^: for instance, by delivering harmonized HBM data for global capacity building and health risk prioritization.
- Occupational Safety and Health Directives: for instance, by informing biological limit values and workplace exposure assessments, and the development of guidance for employers and regulators.
- International Conventions: for instance, by supporting implementation of the Stockholm Convention on Persistent Organic Pollutants (POPs),^3^ Minamata (mercury),^4^ and Rotterdam Conventions^5^ through exposure monitoring and evaluation of control measures.

Alignment with these frameworks will be maintained through active participation in stakeholder consultations, regulatory expert committees, and policy workshops, ensuring that HBM outputs are applicable for regulatory and legislative implementation.

The collaborative efforts between ISES Europe and the OECD have successfully fostered initiatives with significant regulatory relevance in the domains of exposure and effect biomonitoring at a global scale (Table 1). Additionally, the ISES Europe HBM WG efforts have been formally recognized by key regulatory bodies, including the Health Council of the Netherlands, through its development of an assessment framework for biological limit values (https://www.healthcouncil.nl/latest/news/2025/02/25/assessment-framework-for-biological-limit-valuess), and the European Food Safety Authority (EFSA), as reflected in its Roadmap for Action on Risk Assessment of Combined Exposure to Multiple Chemicals (RACEMiC) (de Jong et al., 2022; Lamon et al., 2024). Furthermore, the integration of effect biomarkers into EFSA’s guidance documents underscores the growing regulatory uptake of these scientific advancements.

Engagement with industry has also had tangible impact, leading to European Chemical Industry Council (CEFIC), establishing a dedicated working group to harmonize in-company biomonitoring practices across industry settings and improve data accessibility. This development represents a pivotal step toward aligning industrial practices with scientific and regulatory standards, promoting transparency, and increasing the utility of HBM data to improve workplace safety and advance public health outcomes.

We aim to establish regular communication with policymakers and government agencies, participate in public consultations, provide expert testimony, and offer evidence-based recommendations to leverage public and occupational policies. Concise and informative policy briefs and fact sheets, that summarize key research findings and their implications for human health, will be developed by the working group to inform and influence evidence-based decision-making (Box 3).

This approach will also be adopted by African partners through the collaboration between the African network and the HBM Global Network, within the scope of the BIONET project. Through partnerships, we promote mutual learning, build capacity in occupational and environmental HBM, and strengthen regional data sharing frameworks. This “triangular collaboration” between Europe, Africa, and global partners creates a continuous feedback loop, enabling strategies and infrastructure to adapt based on shared experience.

#### b. Expanding HBM for emerging exposure challenges

In addition to monitoring established exposure pathways, the HBM Global Network will systematically identify and address emerging exposures driven by a complex interplay of environmental, technological, economic, and societal factors. Climate change is reshaping chemical exposure profiles by influencing environmental fate and transport, altering agricultural and industrial practices, and increasing the frequency and severity of extreme weather events. These changes are expected to result in both novel and intensified human exposures (Bhutta et al., 2025; Zare Jeddi et al., 2022a).

To proactively address the evolving risks posed by climate change and other environmental stressors, the HBM Global Network recognizes the need to define strategic priorities for climate-resilient biomonitoring. Although formal plans have not yet been established, existing frameworks such as the NIH Climate Change and Health Initiative (NIH, 2025) and the WHO’s operational guidance (WHO, 2023a) offer valuable foundations for future development. By drawing on these resources, the HBM Global Network can begin to explore how HBM protocols might incorporate climate vulnerability indicators, event-specific metadata, and rapid-response capabilities for post-disaster assessments. Additionally, aligning biomonitoring efforts with environmental observation systems and prioritizing chemicals associated with climate adaptation and mitigation technologies will be essential. This forward-looking approach will enable the dynamic evolution of HBM priorities, ensuring that biomonitoring remains scientifically robust, policy-relevant, and responsive to the complex challenges of a changing global environment.

#### c. Innovation for policy impact through FAIREHR: Future perspective

A key enabler of this vision is FAIREHR (Galea et al., 2025; Zare Jeddi et al., 2023), which serves as a digital infrastructure supporting both the HBM Global Network and the ISES Europe HBM WG. The FAIREHR initiative is uniquely positioned to drive innovation at the science-policy interface by combining the principles of FAIR data management with advanced AI-enabled systems. These capabilities significantly enhance the utility, scalability, discoverability, and responsiveness of HBM and environmental health data for policy development.

FAIREHR will post relevant policy needs, helping researchers and funders to focus on the crucial knowledge gaps.

The planned integration of AI tools within FAIREHR will enable the auditing of study protocols and the identification of inconsistencies or missing metadata, thereby improving the quality and reproducibility of research. This would strengthen the evidence base for policy decisions and advance public trust in regulatory science.

Additionally, AI algorithms within FAIREHR will automatically scan, classify, and synthesize metadata from registered studies. This enables rapid identification of knowledge gaps, emerging exposure trends, and underrepresented populations. Policymakers could use these insights to prioritize research funding, regulatory reviews, and public health interventions.

FAIREHR’s AI systems will leverage machine learning techniques to identify patterns and correlations across diverse datasets including HBM (exposure and effect when available), environmental exposures, demographic profiles, and behavioral factors. By training models on historical and current data, AI can, for instance, identify high-exposure subgroups, optimize sampling strategies, and highlight unexpected exposure pathways. This can help to prioritize populations for biomonitoring campaigns based on predicted high exposure, thus optimizing resources and reducing unnecessary sampling of low-exposure groups. This approach supports the exposome paradigm, but adds a predictive, model-based HBM layer that can be especially useful for data-poor substances or chemical mixtures, where traditional monitoring is limited. AI can also help integrate ingestion-related exposures (e.g., food, water) with environmental contamination and exposure (e.g., air, soil). The latter are often treated separately, despite their associations. These models could simulate future scenarios, such as climate-related disasters or industrial transitions, and inform adaptive policy responses needed. While these predictions are probabilistic and not diagnostic, they provide valuable insights for risk assessment, policy planning, and targeted interventions.

Both ISES Europe HBM WG and HBM Global Network operate in synergy, aligned through shared vision, coordinated governance, and the collaborative platform, FAIREHR.

## 4. Conclusions and call to action

The HBM Global Network has demonstrated the power of coordinated interdisciplinary collaboration to improve the quality, comparability, and policy relevance of HBM data. Progress to date, ranging from harmonized metadata standards and BASIC Guides to the launch of FAIREHR and international capacity building projects has laid the foundation for a truly global HBM ecosystem. The HBM Global Network offers a sustainable framework for collaboration that is not bound by fixed project timelines. It enables ongoing engagement between international experts and professionals, ensuring long-term knowledge exchange, methodological harmonization, and capacity building that extends beyond the lifespan of individual projects.

To shape the future of HBM, we now set measurable goals for 2030:

- **Global Reach**: Strengthen the inclusivity of the HBM Global Network by encouraging participation from a diverse range of countries, ensuring balanced involvement from high-, middle-, and low-income regions. The network is a priority-based and need-oriented collaborative coalition of organizations and experts working together to advance human biomonitoring globally; participation does not require formal membership or dues.
- **Harmonization**: Promote the adoption of core HBM metadata standards in national and regional HBM programs, while aligning with and contributing to complementary efforts by initiatives such as the International Human Exposome Network (IHEN) (Maitre et al., 2024), PARC (Marx-Stoelting et al., 2023), and others working toward interoperable and consistent data frameworks for human exposure and biomonitoring.
- **Guidance Expansion**: Development of at least 15 new chemical-specific BASIC Guides covering environmental and occupational chemicals. The FAIREHR platform hosts the official BASIC Guides. An example of a finalized and publicly available BASIC Guide can be found on the FAIREHR platform for benzene, titled “BASIC Guide: Biomonitoring and Surveillance of Chemical Exposure in Occupational Settings- Benzene” (version 6, January 2025) (https://fairehr.com/BasicGuides).
- **FAIREHR use and progress**: Further advance the FAIR implementation in the FAIREHR registry to increase FAIR compliance of registered HBM studies in FAIREHR. Some other actions include (i) ensure that by 2027, ≥50 % of Network-affiliated studies and projects will be preregistered on FAIREHR with MIR-HBM-compliant metadata, (ii) publish the protocols of the preregistered studies applying tiered access (open or controlled) proportionate to sensitivity; and (iii) ensure MIR-HBM checklists and machine-readable templates will be provided on FAIREHR to improve findability and reuse.
- **Innovation Uptake**: Integrate AI/ML-enabled analytics into multi-national HBM projects by employing machine learning tools to identify complex patterns in exposure data and by expanding the deployment of mobile, low-cost sampling technologies (Chen et al., 2024; Flynn and Chang, 2024; Lazaros et al., 2025; Ozobu et al., 2025; Qureshi et al., 2023; Richardson et al., 2021). These innovations facilitate decentralized, citizen science-driven biomonitoring and accelerate data interpretation, enabling timely and evidence-based policy translation.

The HBM Global Network’s overarching purpose is to advance protection of workers and the general population by reducing exposures to hazardous chemicals, and this goal governs all activities regardless of stakeholder background (Box 4). We invite researchers, regulators, and stakeholders to actively engage with this vision by joining the HBM Global Network (via https://www.fairehr.com), contributing data and expertise, advocating for HBM integration into national frameworks, and co-developing solutions to emerging chemical exposure challenges. Together, we can ensure that HBM becomes a cornerstone of 21st-century environmental and occupational health, supporting a safer and healthier future for all.

## Figures and Tables

**Table 1:** Activities and Expected Impact of some of the Key ISES Europe HBM WG.

Activity		Objective	Expected impact	Status

1	Minimum Information Requirements for Human biomonitoring (MIR-HBM)	Improve data quality, harmonization, reusability, and interpretability. MIR-HBM aims to promote the utility and impact of this research field for public health and occupational safety.	Enhance consistency and comparability of HBM studies and programs, influencing data quality, policy integration and regulatory use.	Completed (Zare Jeddi et al., 2025)
2	FAIR Environmental Health Registry (FAIREHR)	Provide a web-based platform to preregister studies, improve data quality, foster collaboration and ensure efficient dissemination of research findings to policymakers and other stakeholders by providing a unified view of the global research records. FAIREHR promotes transparency, reproducibility, and comparability in scientific research.	Enhance international research collaboration and influence global data sharing practices and regulatory frameworks.	https://www.Fairehr.com The beta version of the platform is now live, and pilot registration for projects and studies has commenced (Galea et al., 2025; Zare Jeddi et al., 2023; Zare Jeddi et al., 2021)
3	OECD Occupational Biomonitoring Guidance	Derive occupational biomonitoring levels (OBLs) to utilize HBM for chemical exposure assessment. Promote harmonized implementation of BM programs.	Support widespread adoption of OECD standards, resulting in harmonized global practices, improve worker health protection and strengthen regulatory risk management.	Milestones achieved (Hopf et al., 2025; Hopf et al., 2024; OECD, 2022)
4	OECD effect biomonitoring guiding principles	Guide the use of effect biomarkers in mixture risk assessment.	Strengthening scientific rigor in chemical mixtures risk assessments, and foster international regulatory adoption.	Completed: (OECD 2025a; OECD 2025b)
5	ISO (International Organization for Standardization) project-Developing guidance on exposure information quality principles for occupational Human biomonitoring	Develop quality principles for occupational exposure data including HBM, adapting EN689 for HBM.	Improve regulatory acceptance and implementation of HBM in occupational settings.	Ongoing activity, expected to be finalised by the end of 2028.
6	BASIC Guides	Harmonize HBM practices among occupational health professionals. BASIC Guide outlines biomonitoring protocols and best practices for a specific chemical, covering aspects such as the identification of relevant exposure biomarkers, procedures for collecting, handling, and analysing biological samples, and the communication of results.	Empower Occupational Health and Safety Professionals (OHPs) to confidently plan and execute BM programs. Improve reliability and comparability of occupational exposure data.	BASIC Guide work is ongoing (Zare Jeddi et al., 2026)
7	HBM and next generation risk assessment	Guidance on integrating HBM along with toxicokinetic, Physiologically Based Kinetic (PBK) modelling into next Generation Risk Assessment (NGRA) framework. This integration addresses aggregate and cumulative exposure and chemical interactions, thereby reducing uncertainties in risk assessment.	Improve sampling strategies and enable more accurate, comprehensive risk evaluations.	Ongoing activity (Reale et al., 2024)
8	HBM role in European REACH regulation and evaluate its impact- State of play and way forward	Demonstrate HBM’s role in policy development and effectiveness assessment, provide recommendations and suggestions for the greater integration of HBM into the regulatory process.	Provide evidence of HBM’s impact on chemical regulation and public health outcomes.	Finalised (Viegas et al., Submitted)
9	Ethical consideration and expanding access to HBM Data (HBM data is distinct from medical data)	Address and provide practical guidance for ethical issues in data protection, consent, responsible data reuse and results communication.	Increase public trust and acceptance; increase study participation; enable responsible sharing/FAIRness without compromising privacy; ensure clear communication of results and related uncertainties	Ongoing activities, Guidance note on ethical aspects and checklist to be published by Q2 in 2026; templates to be hosted on FAIREHR. Guidance note on expanding data access is under development (Hopf et al., 2025; Hopf et al., 2026)
10	A guidance on the use of Artificial Intelligence (AI)/Machine Learning (ML) in HBM studies	Define responsible good practices for AI/ ML in HBM	Advance global standardization and responsible integration of AI/ML methodologies,	Ongoing, expected to be finalised by the end of 2028.

## Data Availability

No data was used for the research described in the article.

## Box 1 Join the HBM Global Network!

We are expanding our Human Biomonitoring (**HBM**) **Global Network** to strengthen global coverage and harmonization. The HBM Global Network’s core principle is health protection, and that regulators, academia, and industry participate together precisely to ensure robust, feasible, and protective practices. The HBM Global Network operates primarily through in-kind/pro-bono contributions from participating experts, institutions, and organizations.

### Priority regions:

- **Oceania:** Australia, New Zealand
- **Latin America:** Mexico, Chile, Argentina, Colombia, Peru, Uruguay
- **Africa:** South Africa, Kenya, Ghana, Senegal
- **Middle East, North Africa (MENA), & Asia:** Egypt, Tunisia, Bangladesh, Pakistan, Central Asia, South Asia, East Asia, Caribbean

### Why join?

- Collaborate with 70+ experts worldwide
- Shape global HBM standards and policy integration
- Access shared protocols, data comparability tools, and training

**Interested? Contact us at:** https://www.fairehr.com; info@fairehr.com

## Box 2 Core principles of the HBM Global Network initiative:

1. Facilitate peer-to-peer exchange of protocols, tools, quality assurance practices, and lessons learned from national and regional HBM programs.
2. Support capacity building in countries or regions where HBM is still emerging, through training, mentoring, and access to validated methods.
3. Promote harmonization of sampling strategies, analytical methods, and data interpretation to enable comparability across regions.

Connect existing initiatives to ensure coherence and minimize redundancies.

## Box 3 Dissemination and audience.

The HBM Global Network is committed to ensuring that its products and deliverables reach a broad and relevant audience to maximize impact and uptake. Dissemination efforts will be tailored to the nature of each output and the needs of key stakeholder groups, including:

### • Policy Makers and Regulators

Deliverables such as guidance documents, BASIC Guides, and policy briefs will be shared with national and international regulatory bodies (e.g., WHO, OECD, UNEP, ILO, EU agencies) through formal channels, stakeholder meetings, and targeted communications. These materials will support evidence-based decision-making and regulatory integration of HBM data.

### • Scientific and Research Communities

Peer-reviewed publications, technical reports, and methodological tools will be disseminated via academic journals, international conferences, webinars, and collaborative platforms. The FAIREHR platform (www.fairehr.com) will serve as a central hub for preregistered studies, protocols, and open-access resources, promoting transparency and reproducibility.

### • Occupational Health and Hygiene Professionals and Practitioners

Practical tools such as the BASIC Guides and training curricula will be distributed through professional networks, workshops, and regional HBM hubs. These resources aim to support the implementation of biomonitoring in workplace settings and enhance data quality and comparability.

### • Low- and Middle-Income Country Stakeholders

Capacity-building materials, multilingual resources, and virtual mentoring opportunities will be made available through the FAIREHR platform and regional hubs. These efforts will support equitable access to HBM knowledge and tools.

### • General Public and Civil Society

Public-facing materials, including infographics, summaries, and educational content, will be developed to raise awareness of chemical exposure and the role of HBM. These will be shared via social media, public webinars, and community engagement initiatives.

All deliverables will be catalogued and accessible through the FAIREHR platform (https://www.fairehr.com), which will serve as the central repository for HBM Global Network outputs. This approach ensures transparency, accessibility, and sustained engagement across sectors and regions

## Box 4 The HBM Global Network governance.

The primary and non-negotiable objective of human biomonitoring is the protection of human health both workers and the general population. This is the shared foundation for all members of the HBM Global Network, irrespective of their affiliations.

Including regulators, scientists, and industry representatives in one forum is not a dilution of this mission but rather a strength. Regulators bring knowledge of legal and policy frameworks; academics ensure scientific rigor and innovation; and industry contributes practical exposure data, technical expertise, and resources that may otherwise be inaccessible. However, participation is not on behalf of institutions or companies but as individual experts.

We adhere to several principles to safeguard health as the overriding priority:

1. **Consensus-based decision-making**: All outputs (guidance notes, protocols, position statements) are developed through collective discussion. If consensus cannot be reached, outputs are not published under the Network’s name.
2. **Transparency**: Affiliations of all members are openly declared, and contributions are documented.
3. **Prioritizing of health protection**: Discussions and outputs are explicitly framed around reducing exposures and protecting health. Economic considerations may inform feasibility, but they do not override health-based objectives.
4. **Complementary expertise**: Experience shows that when all three perspectives, scientific, regulatory, and practical (including industry) are considered together, the resulting guidance is more robust, realistic, and implementable. For example, BASIC Guides and OECD occupational biomonitoring guidance have benefited from such cross-sectoral collaboration, ensuring they are scientifically sound, protective of health, and feasible for workplaces.

In short, the common denominator uniting all Network members is the commitment to protecting human health through biomonitoring. Bringing different perspectives to the table strengthens the ability of HBM to achieve this mission and avoids fragmented approaches.

## Footnotes

1 Council and Parliament agree on simplified and more efficient handling of chemical assessment data – Consilium.

2 https://www.who.int/teams/environment-climate-change-and-health/chemical-safety-and-health/chemicals-road-map.

3 https://www.pops.int/.

4 https://minamataconvention.org/en.

5 https://www.pic.int/.
